# *Staphylococcus saprophyticus* Proteomic Analyses Elucidate Differences in the Protein Repertories among Clinical Strains Related to Virulence and Persistence

**DOI:** 10.3390/pathogens9010069

**Published:** 2020-01-19

**Authors:** Karla Christina Sousa Silva, Lana O’Hara Souza Silva, Guilherme Algusto Alves Silva, Clayton Luiz Borges, Evandro Novaes, Juliano Domiraci Paccez, Wagner Fontes, Marcia Giambiagi-deMarval, Célia Maria de Almeida Soares, Juliana Alves Parente-Rocha

**Affiliations:** 1 Laboratório de Biologia Molecular, Instituto de Ciências Biológicas, Universidade Federal de Goiás, Goiânia-GO 74690-900, Brazil; karlabio@live.com (K.C.S.S.); lanaohara.loss@gmail.com (L.O.S.S.); algustoguilherme1@gmail.com (G.A.A.S.); clayton@ufg.br (C.L.B.); julianopaccez@gmail.com (J.D.P.); cmasoares@gmail.com (C.M.d.A.S.); 2 Departamento de Biologia, Universidade Federal de Lavras, Lavras 37200-900, Brazil; evandro.novaes@ufla.br; 3 Laboratório de Química de Proteínas, Instituto de Biologia, Universidade de Brasília, UnB-Brasilia 70910-900, Brazil; wagnerf@unb.br; 4 Laboratório de Microbiologia Molecular, Instituto de Microbiologia Prof. Paulo de Góes, Universidade Federal do Rio de Janeiro, Rio de Janeiro-RJ 21941-902, Brazil; marciagm@micro.ufrj.br

**Keywords:** proteome, proteomic flexibility, virulence, urease, thioredoxin, biofilm

## Abstract

*Staphylococcus saprophyticus* is a Gram-positive and coagulase negative cocci that composes the skin microbiota and can act as an opportunistic agent causing urinary tract infections, being more frequent in sexually active young women. The ability of a pathogen to cause infection in the host is associated to its ability to adhere to host cells and to survive host immune defenses. In this work, we presented the comparative proteomic profile of three *S. saprophyticus* strains. It was possible to characterize differences in the proteome content, specially related to expression of virulence factors. We compiled this data and previous data and we detected one strain (9325) possessing higher production and secretion of proteins related to virulence. Our results show that phenotypic, genotypic, and proteomic differences reflect in the ability to survive during interaction with host cells, since the 9325 strain presented a higher survival rate after macrophage interaction. In counterpart, the 7108 strain that possesses lower content of proteins related to virulence presented higher ability to form biofilm suggesting that this strain can be better adapted to persist in the host and in the environment. Our work describes, for the first time, proteomic flexibility among *S. saprophyticus* strains, reflecting in virulence and persistence.

## 1. Introduction

The Gram-positive and coagulase negative cocci *Staphylococcus saprophyticus* is the causative agent of urinary tract infections (UTI), being the more frequently causing infection in sexually active young women [1]. The periurethral region can be a repository of this species, as well as skin and mucosal regions [2]. *S. saprophyticus* can compose the skin microbiota and act as opportunist bacteria. Studies with contact sports athletes demonstrates high *S. saprophyticus* prevalence in the skin, suggesting contact among athletes can function as bacteria spreader [3]. *S. saprophyticus* can possess a polysaccharide capsule that enhances virulence in an animal model but does not enhance the internalization rate in human bladder cells [4]. In addition, non-capsular strains have been less frequently isolated from clinical strains (around 1.3%) [5], suggesting the capsule is not required to cause infection.

The ability of *S. saprophyticus* to cause infection can be attributed to virulence factors, such as urease [4,5] surface proteins [6] and D-serine-deaminase protein (DsdA) [7]. Urease was the first virulence factor described in *S. saprophyticus.* Studies performed with urease inhibitors showed that inhibition of urease activity can delay *S. saprophyticus* growth in artificial urine medium, suggesting that urease inhibitors can be used for treatment of UTI caused by this pathogen [5]. Comparative analysis using a genomic approach describes that *S. saprophyticus*, compared to other coagulase negative *Staphylococcus* species, lacks many of the adhesion proteins and other virulence factors that can explain differences at a clinical level [8]. In counterpart, *S. saprophyticus* presents importance to public health not only by the ability to cause human infections, but also by the ability to persist in the environment, acquire and transmit plasmids that can confer antibiotic resistance [9,10].

The *Staphylococcus* species can form biofilms dependent on polysaccharide intercellular adhesin (PIA), synthesized by *ica* operon [11]. Biofilms can reduce access of the host defense system to *Staphylococcus* and impair antibiotic action. Also, conjugation can occur at higher levels in staphylococcal cells in biofilms compared to planktonic cells [12]. Analysis of 169 *S. saprophyticus* strains shows that 70% of these strains possess ability to form biofilm. In addition, the biofilm formation increases the resistance to five antibiotics (vancomycin, oxacillin, trimethoprim/sulfamethoxazole, ciprofloxacin, and norfloxacin) in around 32-fold [13].

Genotypic variation among *S. saprophyticus* strains has already been described. Studies performed with 236 *S. saprophyticus* strains obtained from patients demonstrated that 100% of the strains possess genes encoding virulence factors, such as urease, DsdA, uroadherence factor a (UafA), and autoloysins (Aas), suggesting that these genes are required for infection. In counterpart, the gene encoding the surface protein SdrI is detected only in 10% of the strains, suggesting it is not an essential gene for infection [14]. A previous work by our research group analyzed comparative proteomic data among capsular and non-capsular *S. saprophyticus* strains, elucidated the *S. saprophyticus* exoproteomic repertoire, and showed that different strains possess different secreted machinery that can be used during infection. For example, the highly capsular 9325 strain secretes higher content of antigenic proteins and transglycosylases while the non-capsular 7108 strain does not secrete the SsaA antigenic protein and secretes higher content of proteases. These results show diversity in protein secretion among strains [15].

In our work, we used a proteomic approach to compare three *S. saprophyticus* strains possessing different patterns of the capsule: the reference strain ATCC 15305 possessing capsule, the non-capsular 7108 strain, and the highly capsular strain 9325. Of special note, ATCC 15305 produces higher content of proteins for purine biosynthesis. On the other hand, 9325 secretes higher level of urease. The production of thioredoxin, related to oxidative stress response, is also different among the strains. Our results show that these strains use different molecular machineries that can confer the ability to cause infection. The ability to produce, secrete, and use virulence factors is deeply related to the bacterial survival during infection and each strain can use a different repertoire, related to its metabolic flexibility to cause infection in the host.

## 2. Results

### 2.1. Evaluation of S. saprophyticus Strains Cell Growth

In order to perform the comparative proteomic analyses using cells from the *S. saprophyticus* strains at the same point of the cell growth curve, we performed the evaluation of cell growth using the spectrophotometer, as described above. The result is shown in Figure S1. The result shows that all the strains present the same profile of cell growth during 10 hours. Previous cell growth analysis during 18 hours also shows these strains possess similar cell growth curves [15]. We speculate that the capsule size can interfere in the optical density and can explain the differences shown in Figure S1.

### 2.2. Proteomic Profile of S. saprophyticus Strains

Protein extracts were obtained from three biological replicates. Trypsin digested protein extracts were quantified by Nano-UPLCMS^E^ and protein and peptide data were generated by PLGS. A total of 276 proteins were detected in this work. The proteins identified were functional categorized and description of the identified proteins is shown in Table S1. The FDR was obtained for all replicates. This strategy resulted in replicate 1, 2, and 3 rates of 1.86%, 0%, and 0.46% for ATCC 15305, 0.89%, 2.38%, and 0.57% for 7108; and 1.94%, 0.70%, and 0.70% for 9325, respectively. The number of identified peptides was 14,384; 18,995 and 26,328 in ATCC 15305; 14,244; 15,438 and 21,854 in 7108; and 12,890; 17,785 and 19,055 in 9325 in replicate 1, 2, and 3, respectively. Regarding peptides parts per million errors (ppm), the majority (77.8%, 76.0%, and 75.0% for ATCC 15305; 78.5%, 77.4%, and 76.1% for 7108; and 77.4%, 74.7%, and 75.9% for 9325 in replicate 1, 2, and 3, respectively) was detected with an error of less than 10 ppm. Dynamic range detected a 3-log range concentration and a good distribution of high and low molecular weights in all samples (Figure S2).

Among the non-regulated proteins identified in the three *S. saprophyticus* strains, it was possible to detect proteins related to the transcription and translation processes, glycolysis pathway, and amino acid metabolism. A total of 170 proteins were identified as non-regulated among the strains (Table S1).

### 2.3. Differentially Abundant Proteins Among S. saprophyticus Strains

In order to detect differentially abundant proteins among the three *S. saprophyticus* strains, we analyzed the proteins presenting statistically significant differences in abundance values among the strains. It was possible to detect proteins related to virulence and host pathogen interaction differentially regulated among the strains. Of special note, we selected proteins related to oxidative stress, the urease system, and *de novo* purine biosynthetic process. These proteins are listed in Table 1.

**Figure 1:**
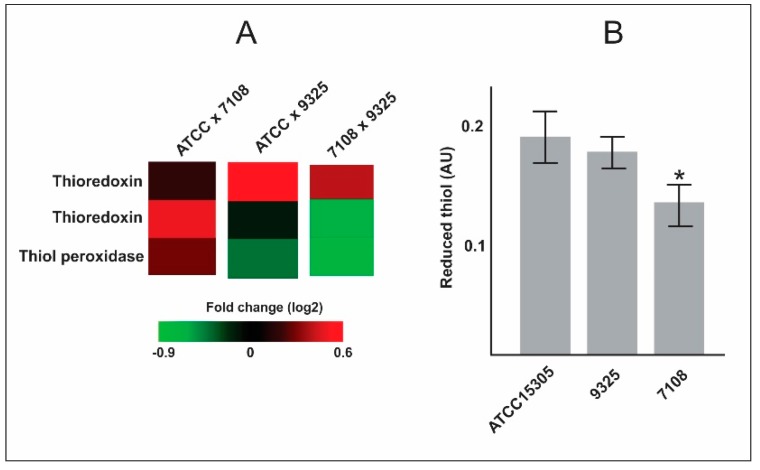
Thioredoxins and thiol peroxidases expression and enzymatic assay. (**A**) Heat map of protein abundance. Heat map showing fold change (log2) comparing proteomic data of thioredoxins and thiol peroxidase abundance among *S. saprophyticus* strains. (**B**) Enzymatic assay of thiol reduction. The reactions were performed using protein extracts from the three *S. saprophyticus* strains. Reduced thiol formed was measured. The assay was performed in biological duplicate and experimental triplicate. Asterisk indicates statistical significance (*p* < 0.05) when compared to any of the other strains using Student’s t test.

We detected thioredoxins less abundant in the 7108 strain when compared to ATCC 15305 and 9325 strains. The abundance of thioredoxin was not statistically different when comparing the ATCC 15305 to 9325 strains. In order to validate the proteomic data, we performed the thioredoxin assay, as described above. The result of the enzymatic assay corroborates our proteomic analysis (Figure 1), showing a lower level of thioredoxin abundance and activity in the strain 7108.

**Figure 2:**
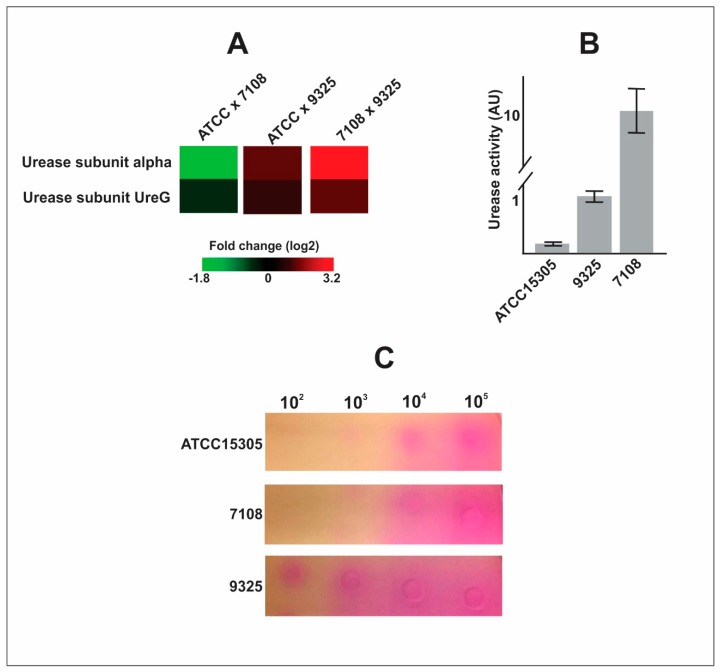
Urease enzymatic assays with protein extract from and secreted by *S. saprophyticus* cells. (**A**) Heat map of urease subunits expression. Heat map showing fold change (log2) comparing proteomic data of urease subunits abundance among *S. saprophyticus* strains. (**B**) Urease activity. The enzymatic assay is shown in arbitrary units (AU) performed with protein extracts of *S. saprophyticus* strains. The experiment was performed using three biological replicates and with three technical triplicates. (**C**) Evaluation of secreted urease activity. The *S. saprophyticus* cells were serially diluted from 10^5^ to 10^2^ cells and inoculated in urease agar plates. The urease activity is detected by change of color of the medium, from yellow to purple. The experiment was performed with biological triplicates and representative images are shown.

The 7108 *S. saprophyticus* strain presented a very expressive amount of two subunits from the urease system, compared to the strains 9325 and ATCC 15305. The strain presenting the smallest content of these proteins was the ATCC 15305 strain (Figure 2A). In order to validate this finding, we performed the urease enzymatic assay in the protein extracts from the three analyzed strains. The result depicted in Figure 2A corroborates the findings of the proteomic analysis. The strain 7108 presented very increased urease activity when compared to the other strains. Comparison among 9325 and 15305 strains shows that the first one presents a higher level of urease activity. Since urease is a secreted protein, we decide to investigate the urease activity outside the cell. We performed the urease activity assay of secreted urease in agar plates. The *S. saprophyticus* cells were serially diluted (10^2^ to 10^5^ cells) and inoculated in urease agar plates. The result is shown in Figure 2C. The results show that the ATCC 15305 presents low urease activity inside (0.1 AU/assay) and outside the cell (detected only in 10^4^ and 10^5^ cell dilutions), reflecting probably a lower production of the enzyme. Although the 7108 strain presents high urease activity inside the cell, the urease activity outside the cell is low (detected only in 10^4^ and 10^5^ cell dilutions), suggesting this enzyme is accumulating in the cytoplasm and is not efficiently secreted by this strain. In counterpart, the 9325 strain does not accumulate urease inside the cell and the secretion system is more efficient, since the urease activity inside the cell is low and the urease activity outside the cell is very high, being detected in all the cell dilutions tested.

**Figure 3:**
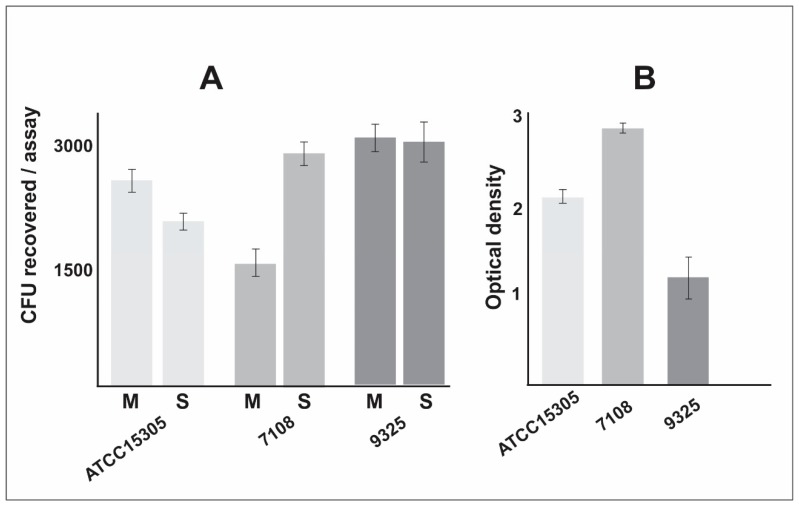
*S. saprophyticus* interaction assay with macrophages and evaluation of biofilm formation. (**A**)The *S. saprophyticus* cells were incubated with macrophages and, after interaction assay, the supernatant (S) containing non-phagocyted bacterial cells were plated in BHI medium. The macrophages were lysed and colony-forming unit (CFU) recovered and plated in BHI medium (M). The experiments were performed in biological triplicate and standard error of the mean was calculated. (**B**) The biofilm assay was performed in a polystyrene plate, cells were fixed and stained with crystal violet. Optical density was measured at 570 nm wavelength. The experiments were performed in biological triplicate and standard error of the mean was calculated.

The *S. saprophyticus* strains used in this work were previously analyzed and also differ in the exoproteome content [15]. In order to compare if the phenotypic, exoproteomic, and proteomic differences described for these strains could reflect the ability to survive during interaction with host cells, we performed interaction assays of *S. saprophyticus* cells with macrophages. The result is shown in the Figure 3A. It is remarkable that the 9325 strain presents the highest ability to survive during interaction with macrophages, when compared to ATCC 15305 and 7108 strains. The strain presenting the lowest survival rate to macrophage interaction was 7108. The ATCC 15305 survival rate is slightly reduced in comparison with the 9325 strain. We also evaluated the ability of the *S. saprophyticus* strains to form biofilm in polystyrene plates. The result is shown in Figure 3B. It was possible to detect that the non-capsular strains 7108 possess the higher ability to form biofilm when cultured in BHI medium containing 1% glucose.

**Figure 4:**
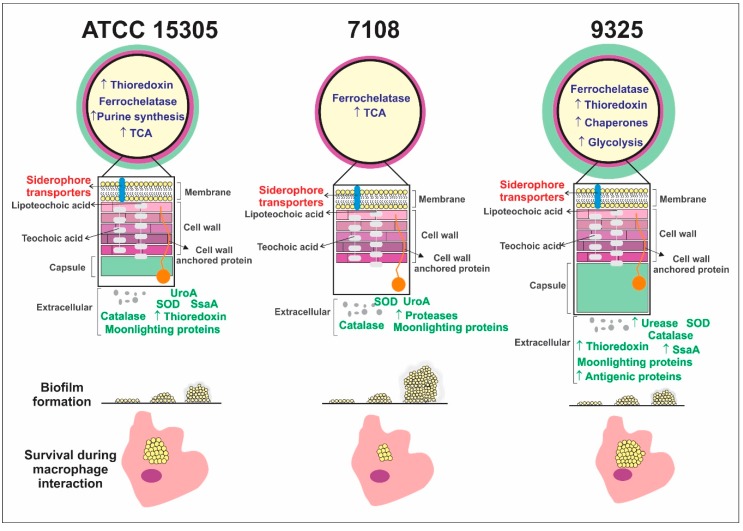
Schematic representation of phenotypic, exoproteomic, and proteomic differences among the *S. saprophyticus* strains analyzed. Proteins and phenotypic differences (detected in previous works and in this work) related to virulence, pathogenesis, and persistence are shown. TCA: tricarboxylic acid cycle; UroA: uro-adherence factor A; SsaA: staphylococcal antigenic protein A; SOD: superoxide dismutases. Protein names shown in blue correspond to proteins identified in this work. Red protein names correspond to proteins identified by Souza and collaborators [16] and green protein names correspond to proteins identified by Oliveira and collaborators [15]. Blue protein names correspond to proteins identified in this work.

In order to summarize phenotypic, proteomic, and exoproteomic differences related to pathogenesis and virulence among the strains, we schematized the phenotypes and proteins identified in the strains. The scheme is shown in Figure 4. The schematic figure was generated based on previous work of phenotypic characterization of the capsule [14], our previous studies of comparative exoproteome analysis [15], and in the results of this work. It is remarkable that the 7108 strain possesses a reduced repertoire of proteins related to virulence compared to the ATCC 15305 and 9325 strains. The secretion of uro-adherence factor (UroA) and proteases was described in the exoproteome analysis [15]. The ATCC 15305 strain machinery related to virulence and pathogenesis detected in this work includes high production of proteins related to purine synthesis, tricarboxylic acid cycle, and thioredoxins, besides the previous description of secretion of thioredoxins, UroA, and the antigenic protein SsaA [15]. The 9325 machinery involved in virulence seems to be wider when compared to the ATCC 15305 and 7108 *S. saprophyticus* strains. This strain secretes several proteins related to virulence, such as a high amount of urease, SsaA, several antigenic proteins, and thioredoxins [15], and we detected the production of high amounts of thioredoxins, chaperone proteins, and proteins related to glycolysis in comparison with the other strains. Some proteins important during infection, such as ferrochelatases and siderophore transporters, were detected in this work. Previous analysis from our group demonstrated the relevance of iron metabolism to increase the *S. saprophyticus* survival rate during macrophage infection, reinforcing these proteins are important during the infection process [16].

## 3. Discussion

In the last years, proteomic approaches have been used to understand pathogens machinery used to infect the host [17,18]. Specially, staphylococcal species have been studied by these techniques, providing elucidation of processes used during infection and contributing in the discovery of virulence determinants, thus enlarging knowledge about pathogenicity mechanisms [19,20].

The knowledge about the proteomic profile of *S. saprophyticus* is still incipient. Few analyses using a proteomic approach were performed in *S. saprophyticus*, but our previous comparative exoproteome analysis among three *S. saprophyticus* strains (7108 non-capsular strain and ATCC 15305 and 9325 capsular strains) shows diversity in the exoproteome contents, suggesting these strains can possess different machineries of secreted proteins to promote infection [15]. In this work, we are describing the proteome global content of these three *S. saprophyticus* strains. The cell growth curves of these strains were evaluated in rich medium and they presented similar growth rates in this condition (Figure S1). The proteomic approach was used to obtain the proteome profile of the strains and the statistical analysis performed included results obtained from biological triplicates and experimental replicates in order to enhance the resolution of the results obtained. From the total of 276 identified proteins, 170 were not regulated among the strains. They include proteins involved in protein synthesis, transcription, and DNA metabolism (Table S1) and reinforce that *S. saprophyticus* cells from the three strains were grown until the same metabolic moment of the cell growth curve before proteomic analysis.

Among the 106 regulated proteins detected in each *S. saprophyticus* strain, we highlighted proteins related to virulence and pathogenesis (Table 1). We detected that the 9325 and ATCC 15305 strains possess higher amounts of thioredoxins and reductases in comparison with the 7108 strain and this data was confirmed by thiol reduction dosage (Figure 1). Thioredoxin system (Trx)—formed by thioredoxin reductase, thioredoxin, and NADPH—can scavenge ROS [21]. In *S. aureus*, the glutathione system is deficient and Trx system is important for bacterial survival under oxidative stress conditions [22]. In this sense, strains possessing higher content of thioredoxins could be related to higher adaptation and survival under oxidative stress condition. Other difference detected among the *S. saprophyticus* strains is the content of urease. We detected the 7108 strain possesses higher content of urease in the proteomic analysis in comparison with ATCC 15305 and 9325 strains. We checked if the higher urease content could be due to the accumulation of urease inside the cell or could be due to the higher production of this protein in the 7108 strain. The results show that this strain does not secrete urease efficiently and urease remains inside the cell (Figure 2). Urease secretion is considered a secreted virulence factor in *S. saprophyticus*, contributing to urophatogenicity in rats [4] and facilitating *S. saprophyticus* growth in artificial urine medium [5]. This finding suggests strains 9325 and ATCC 15305 could present higher ability to survive during host interaction since they can export urease with more efficiency when compared to the 7108 strain.

Of special note, we detected high abundance of proteins related to purine synthesis in the ATCC 15305 strain. The purine biosynthesis has been associated to intracellular survival of bacterial pathogens, including the uropathogenic *Escherichia coli* [23]. The inactivation of purine *“de novo”* synthesis is also associated to reduction of *E. coli* virulence and cell growth [24]. In the bacteria *Pseudomonas fluorescens*, the disruption of purine biosynthesis operon is associated with the reduction of the ability to form biofilm [25]. Further analyses are required to evaluate the effect of the high abundance of proteins related to purine biosynthesis in the ATCC 15305 strain.

It was possible to note that phenotypic and proteomic differences described among these strains reflect in the survival ability during interaction with host cells (Figure 3A) and in the ability to form biofilm (Figure 3B). Our findings show the *S. saprophyticus* 9325 strain possesses higher survival rate after interaction with macrophages, followed by the ATCC 15305 strain. The lowest survival rate was detected in the 7108 strain. In counterpart, the 7108 presented the highest ability to form biofilm when cultured in BHI medium enriched with glucose. This data corroborates previous results using *Streptococcus pneumoniae* as the model. Experiments of biofilm formation using defected mutants for capsule production describe similar results, suggesting that the capsule is antagonistic to biofilm formation [26].

The differences among these strains related to virulence and pathogenicity described in this work and in previous works [14,15] are summarized in Figure 4 and show that 9325 and ATCC 15305 possess larger amounts of proteins that could enhance pathogenicity and virulence. The lowest machinery of proteins that could enhance virulence is described in the 7108 strain. In counterpart, the ability to form biofilm is increased in the 7108 strain. Although the lower production of virulence factors detected in this and in previous work, the ability to form biofilm can enhance the survival rate of this strain in the host and in the environment. Biofilm formation is associated with reduction of the access of the host defense system to *Staphylococcal* cells and is also associated with impairing of antibiotic action [12]. Analysis of clinical *S. saprophyticus* strains revealed that the biofilm formation ability is presented by around 70% of the strains, suggesting it could be important during infection since it increases resistance to several antibiotics [13]. The increase in the persistence rate caused by biofilm is also important since it enhances conjugation in staphylococcal species [12]. The operon *ica*ABCD presented in staphylococcal species promotes biofilm synthesis [27]. Proteins synthesized by the *ica* operon are not listed among the predicted proteins produced by the *S. saprophyticus* complete genome (ATCC 15305 strain, accession number AP008934 at the NCBI database). However, gene products from this operon have recently been identified, sequenced, and added to the NCBI database (accession numbers WP_048792334, WP_048792335, WP_048792336, SUM81325), which reinforces the hypothesis that this operon is conserved among the species of the genus.

Our results and analyses show that clinical *S. saprophyticus* strains can possess different protein machinery and phenotypic characteristics that can confer ability to invade and persist in the human host. However, further analysis involving other capsular and non-capsular strains can be helpful to enlarge the robustness of these data. These results can reveal important protein targets for drug development, taking into account the conserved machinery among clinical strains. Thus, it is not enough that the strain possesses the genes associated with virulence and persistence, but the gene must be expressed and used at the time of infection in the host to be a good target. In this sense, further studies with mutant for protein targets and search for protein inhibitors can be helpful to validate these data and advance in the characterization of this pathogen, purposing new advances in the treatment of UTI caused by *S. saprophyticus.* This is the first description of proteomic flexibility among *S. saprophyticus* strains reflecting in virulence, pathogenicity, and persistence.

## 4. Material and Methods

### 4.1. S. saprophyticus Strain Maintenance and Culture Conditions

*S. saprophyticus* reference strain ATCC 15305 and the clinical strains 7108 and 9325 (kindly provided by Lennart Marlinghaus from the Department of Medical Microbiology, Ruhr-University Bochum) were used in this study. These strains were previously genotypically and phenotypically characterized. They differ in the presence of capsule (ATCC 15305 possess capsule, 7108 is non-capsular, and 9325 possess a thick capsule) in the presence of virulence factors and in the exoproteome content [14,15,28]. *S. saprophyticus* cells were cultured in BHI medium (Sigma-Aldrich, St. Louis, MO, USA) and stored at −80 °C in 50% (*v*/*v*) glycerol. In order to obtain the protein extracts, a single *S. saprophyticus* colony of each strain was pre-incubated separately in BHI medium until the stationary phase (after around 20 hours) with shaking at 36 °C. After, the inoculum was performed using 1% of the pre-inoculum and the cells were incubated at 36 °C with shaking until the optical density 0.6 at 600 nm wavelength using spectrophotometry SpectraMax Paradigm (Molecular Devices, Lagerhausstrasse, Austria).

### 4.2. Cell Growth Curve of S. saprophyticus Strains

Each strain was cultured for the pre-inoculum and inoculum, as described above. The cell growth curves were performed following the guideline of the Clinical and Laboratory Standards Institute. The experiments were performed in three independent replicates. The initial optical density was adjusted to 0.04 in the BHI medium (wavelength 600 nm at spectrophotometry equipment). The inoculum was split into 200 μL aliquots and placed in 96-well plates and left under agitation at 37 °C. Cell growth was monitored each 2 h for a 10 h period through spectrophotometry using SpectraMax Paradigm at 600 nm wavelength (Molecular Devices, Lagerhausstrasse, Austria). Bacterial cells presenting optical density higher than 0.8 at 600 nm wavelength were diluted prior to optical density measuring.

### 4.3. Obtaining the Proteins Extracts from S. saprophyticus

Protein extracts were obtained by using glass beads 0.2 to 0.8 nm (Sigma Aldrich, St. Louis, MO, USA) to disrupt the cells using bead beater (Biospec, Bartlesville, OK, USA) during three cycles of 30 seconds. After, the protein extract was obtained by centrifugation at 10000× *g* for 15 min. The protein extracts were quantified using Bradford reagent (Sigma Aldrich, St. Louis, MO, USA) using spectrophotometry SpectraMax Paradigm (Molecular Devices, Lagerhausstrasse, Austria). The integrity of the protein extract obtained was evaluated on SDS-PAGE containing 30 μg of extract of each sample. Biological triplicates were obtained and used for proteomic analysis.

### 4.4. Digestion of Protein Extracts for Nano-ESI-UPLC-MS^E^ Acquisition

A total of 150 µg of each protein extract was trypsin digested. The protocol used for digestion was previously described [15]. A total of 20 ng of trypsin (Promega, Madison, WI, USA) was used for each sample. The samples were then treated with trifluoroacetic acid (TFA) 0.1% (Sigma Aldrich, St. Louis, MO, USA) and purified on C18 ZipTips (Millipore, Darmstadt, Germany). The resins were equilibrated with acetonitrile (ACN) prior to use (Sigma Aldrich, St. Louis, MO, USA), followed by washing with the following solutions: (1) 80% ACN and TFA 0.1%, (2) 50% ACN and 0.1% TFA, (3) 30% ACN and 0.1% TFA, and finally (4) 0.1% TFA.

### 4.5. Data Processing and Protein Identification

The obtained raw mass spectrometry data were processed using the ProteinLynx Global Server v3.0.2 (PLGS) (Waters Corporation, Milford, MA, USA). For protein identification, processed spectra were searched against *S. saprophyticus* protein sequences (from Uniprot Proteomes) together with reverse sequences for false positive rate (FDR) estimation. The mass error tolerance for peptide identification was under 50 ppm. Criteria for protein identification included: (i) at least 2 fragment ions per peptide, (ii) at least 5 fragment ions per protein; (iii) at least 1 peptide per protein; (iv) carbamidomethylation of cysteine as a fixed modification; (v) phosphorylation of serine, threonine, and tyrosine, and methionine oxidation as variable modifications; (vi) maximum protein mass of 600 kDa; (vii) 1 missed cleavage site was allowed for trypsin; (viii) maximum of 5% of FDR was allowed. The protein and peptides table generated by PLGS were merged and the dynamic range, peptide detection type, and mass accuracy were determined for each sample using MassPivot v3.1, FBAT, and Spotfire^®^ v7.0.0 program (TIBCO software, Palo Alto, CA, USA), as previously described [29]. Microsoft Excel^®^ (Microsoft) was used for table manipulations. The mass spectrometry proteomics data have been deposited to the ProteomeXchange Consortium via the PRIDE [30] partner repository with the dataset identifier PXD017112.

### 4.6. Statistical Analysis of Proteomic Data

The statistical analysis was conducted using data from biological and experimental replicates. The amount of each protein, from each *S. saprophyticus* strain and replicate, was used as a measure of protein expression, as described previously [15]. The R software was used and the expression data were log_2_ transformed and quantile normalized with the limma package [31], using the normalize between arrays function. Each biological and technical replicate is treated differently by limma. Differential abundance analyses between the three *S. saprophyticus* strains were performed with an empirical Bayes method implemented in the limma package to moderate the standard errors of the estimated log-fold changes [32]. This analysis results in more stable inference and improved power, especially for experiments with small numbers of replicates. Proteins were declared differentially abundant using a threshold of 0.05 false discovery. The functional classification was performed by using Uniprot (http://www.uniprot.org).

### 4.7. Urease Activity Assays

The urease activity was assayed as described [33]. PEB buffer (100 mM sodium phosphate; 10 mM EDTA) was added to the samples containing 10 μg of protein extract and 50 mM urea, heated to 37 °C for 30 min. Then 80 μL alkaline hypochlorite and 80 μL phenol nitroprusside solution were added to the samples and heated to 50 °C for 6 min. The absorbance was read at 625 nm using SpectraMax Paradigm (Molecular Devices, Lagerhausstrasse, Austria). The readings were compared to the standard curve with NH_4_Cl concentrations ranging from 5 μM to 500 μM (5; 10; 25; 50; 100; 150; 200; 150; 300; 500 μM NH_4_Cl) as well as a standard without urea.

Bacterial cells were also cultured on urea agar-based plates, as described by [34]. Cells were plated in different dilutions (10^2^; 10^3^; 10^4^; 10^5^ cells) and kept for 20 h at 37 °C.

### 4.8. Thioredoxin Assay

A total of 10^9^ bacterial cells—counted in cytometry flow—from each strain were lysed with 0.5 ml lysis buffer (50 mM Tris-HCl; 150 mM NaCl; 50 mM EDTA; pH 7.2) and approximately 0.5 g of glass beads. The samples were vortexed for 3 cycles of 3 min with 1-minute interval. Cellular debris were removed by centrifugation. Then, 100 μL of supernatant plus 100 μL 500 mM phosphate buffer (pH 7.5) and 1 mM DTNB (5.5 dithio-bis-(2-nitrobenzoic acid) were added to each sample. The reaction took place at room temperature for 15 min. The absorbance was read at 412 nm using SpectraMax Paradigm (Molecular Devices, Lagerhausstrasse, Austria).

### 4.9. S. saprophyticus Interaction Assay with Macrophage Cells

The interaction assay was performed as previously described [16]. Macrophages J774 1.6 derived from *Mus musculus* (Banco Central do Rio de Janeiro, Rio de Janeiro, RJ, Brazil) were cultured in RPMI medium with 1% amino acid solution and 10% fetal bovine serum. A total of 10^6^ macrophages were placed into 6-wells plates and activated with Interferon-γ 24 h prior to infection to a final concentration of 1 unit/ml. Bacterial cells were cultured in BHI broth to the optical density of 0.2 at 620 nm, harvested through centrifugation (3000 rpm for 5 min), washed with saline solution 0.9%, and resuspended in RPMI medium without antibiotics. The macrophage medium was replaced with the medium containing 50 × 10^6^ *S. saprophyticus* cells. After 2 h infection, 50 μL of supernatant of each well was plated on BHI agar plates in triplicates for each well. Each well was washed three times with 0.9% saline solution and the macrophages lysed for 10 min with 1.5 mL of ice-cold ultrapure water. After lysis, bacterial cells were centrifuged and resuspended in 300 μL 0.9% saline solution and 50 μL plated onto BHI agar plates in triplicates for each well. The plates were kept for 20 h at 37 °C and colony formation units (CFU) counted for analysis.

## 5. Conclusions

- Different bacterial strains from *S. saprophyticus* presenting phenotypic and genotypic variations also present proteome global content.
- The capsular strains ATCC 15305 and 9325 possess higher amounts of thioredoxins and reductases in comparison with the 7108, which could reflect in the ability to combat oxidative stress and to survive during interaction with host cells.
- The capsular strains 9325 and ATCC 15305 are more efficient in exporting urease, which can enhance ability to survive in the presence of urine.
- The non-capsular strain 7108 presented higher ability to form biofilm, which is a particularly important characteristic that enhances the ability to persist in the host and in the environment.
- The proteomic approach can be used to detect and describe proteomic flexibility related to virulence, pathogenicity, and persistence of pathogens.

## 6. Disclosures

This work was supported by Fundação de Amparo à Pesquisa do Estado de Goiás (FAPEG, Pronex) and Conselho Nacional de Desenvolvimento Científico e Tecnológico (CNPq) This work is part of Instituto Nacional de Ciência e Tecnologia da Interação Parasito-Hospedeiro (INCT-IPH). KCSS was supported by scholarship from Universidade Federal de Goiás and LOHSS was supported by scholarship from Coordenação de Aperfeiçoamento de Pessoal de Nível Superior (CAPES).

## Figures and Tables

**Table 1:** Proteins related to virulence differentially expressed among *S. saprophyticus* strains *.

Accession Number ^1^	Protein Description	Log FC ^2^			*p*-Value ^3^		
		ATCC * vs. 7108	ATCC * vs. 9325	7108 vs. 9325	ATCC * vs. 7108	ATCC * vs. 9325	7108 vs. 9325
Oxidative Stress							
Q49WR2	Thioredoxin	0.494	−0.133	−0.627	0.011	0.480	0.001
Q49YE4	Probable thiol peroxidase	0.280	−0.419	−0.699	0.376	0.085	0.005
Q49UT8	Alkyl hydroperoxide reductase subunit C	0.490	−0.408	−0.899	0.026	0.040	0.000
Q49XC1	Catalase	3.691	3.811	0.120	0.000	0.000	0.735
Q49XN4	Peptide methionine sulfoxide reductase MsrB	0.228	0.460	0.232	0.449	0.044	0.339
Q49XZ6	Superoxide dismutase [Mn/Fe]	0.338	−0.406	−0.744	0.128	0.038	0.000
Q49YE0	Putative universal stress protein SSP1056	2.055	2.055	0.000	0.000	0.000	1.000
Q49UU5	Nitronate monooxygenase	−0.339	1.063	1.402	0.402	0.001	0.000
Nitrogen Metabolism							
Q4A0J5	Urease subunit alpha	−1.782	1.338	3.120	0.034	0.068	0.000
Q4A0J8	Urease accessory protein UreG	−0.416	0.936	1.352	0.464	0.038	0.003
*De Novo* Purine Biosynthetic Pathway							
Q49WI9	N5-carboxyaminoimidazole ribonucleotide synthase PurK	2.377	2.377	0.000	0.000	0.000	1.000
Q49WJ0	Phosphoribosylaminoimidazole-succinocarboxamide synthase PurC	2.184	2.184	0.000	0.000	0.000	1.000
Q49WJ1	Phosphoribosylformylglycinamidine synthase subunit PurS	1.035	1.035	0.000	0.000	0.000	1.000
Q49WJ2	Phosphoribosylformylglycinamidine synthase subunit PurQ	1.006	1.006	0.000	0.011	0.007	1.000
Q49WJ3	Phosphoribosylformylglycinamidine synthase subunit PurL	3.345	3.345	0.000	0.000	0.000	1.000
Q49WJ5	Phosphoribosylformylglycinamidine cyclo-ligase PurM	2.365	2.365	0.000	0.000	0.000	1.000
Q49WJ7	Bifunctional purine biosynthesis protein PurH	4.249	4.249	0.000	0.000	0.000	1.000
Q49WJ8	Phosphoribosylamine-glycine ligase PurD	3.038	3.038	0.000	0.000	0.000	1.000

^1^ Accession number provided by Uniprot Database (http://www.uniprot.org/). ^2^ Obtained from limma’s top table by subtracting the average expression in log2 scale against the strains. ^3^ Proteins with *p*-value ≤ 0.05 were considered regulated among the strains. *p*-value from the Student’s t distribution. * ATCC 15305 strain.

## Supplementary Materials

The following are available online at https://www.mdpi.com/2076-0817/9/1/69/s1, Figure S1: *saprophyticus* cell growth curve. The graphic demonstrates *S. saprophyticus* strains growth behavior in 10h period. Figure S2: Quality analysis of proteomic data from *S. saprophyticus* ATCC 15305, 7108 and 9325. Table S1: List of proteins identified in this work.
