# Supplementary material for: Staphylococcus saprophyticus Proteomic Analyses Elucidate Differences in the Protein Repertories among Clinical Strains Related to Virulence and Persistence
Source: Pathogens. 2020 Jan 19;9(1):69. doi: 10.3390/pathogens9010069 (PMC7169411; doi:10.3390/pathogens9010069)
Supplement: Supplementary file 1 [file pathogens-09-00069-s001.zip › New folder/Figure S1.pdf]

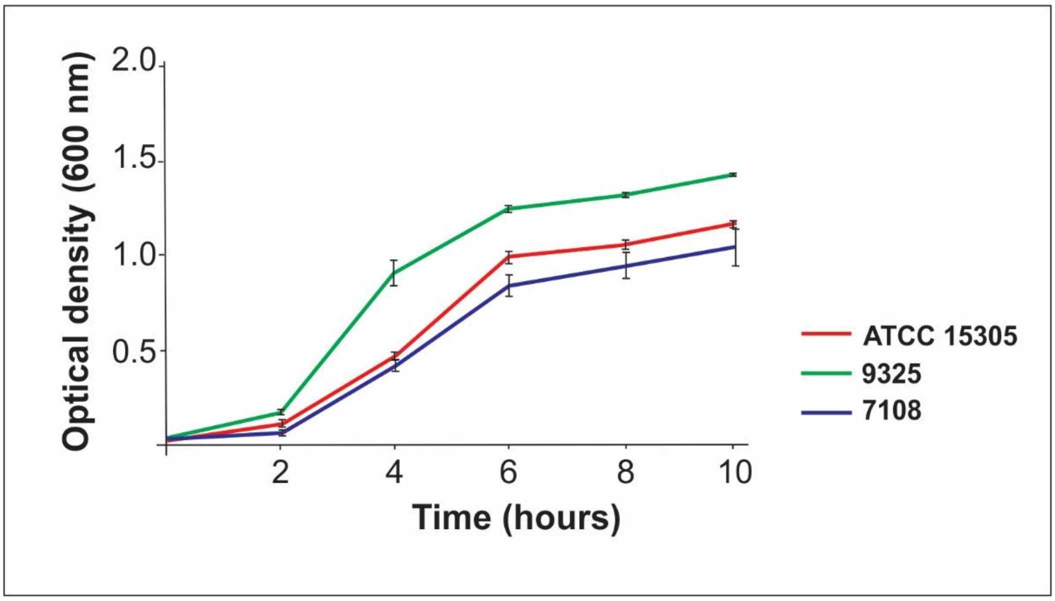

Supplementary Figure 1 – *S. saprophyticus* cell growth curve. The graphic demonstrates *S. saprophyticus* strains growth behavior in 10h period.
