# Supplementary material for: Staphylococcus saprophyticus Proteomic Analyses Elucidate Differences in the Protein Repertories among Clinical Strains Related to Virulence and Persistence
Source: Pathogens. 2020 Jan 19;9(1):69. doi: 10.3390/pathogens9010069 (PMC7169411; doi:10.3390/pathogens9010069)
Supplement: Supplementary file 1 [file pathogens-09-00069-s001.zip › New folder/Figure S2.pdf]

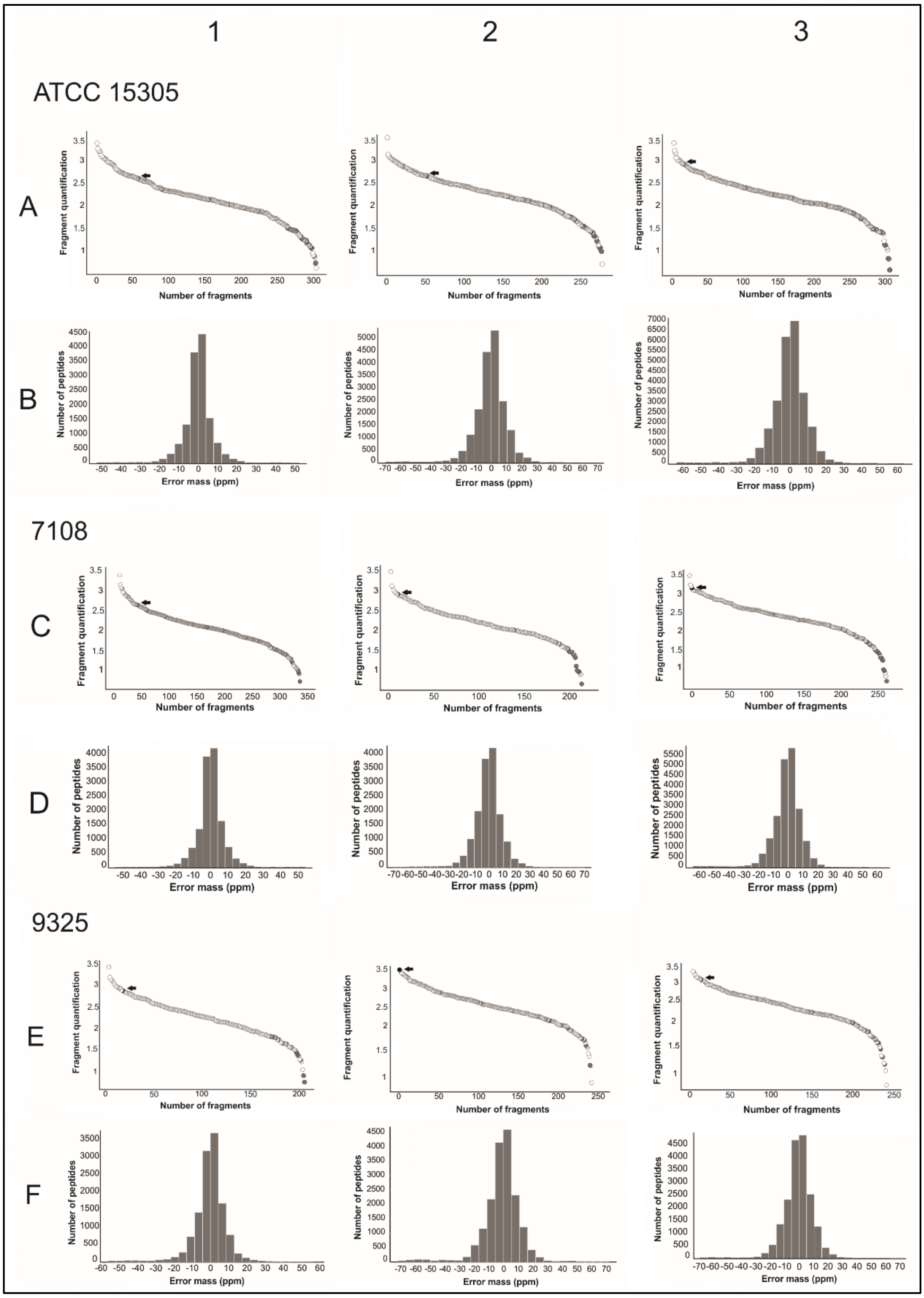

Supplementary Figure 2 – Quality analysis of proteomic data from *S. saprophyticus* ATCC 15305, 7108 and 9325. 1,2 and 3 represents replicates 1, 2 and 3 respectively. Detection of dynamic range of proteomic analysis. Protein data were used to calculate the order of magnitude of protein identification. Regular, reverse and standard proteins are represented in blank, grey and black (arrow), respectively. Regular and reverse proteins indicate identified proteins using regular and reverse proteome of *S. saprophyticus*. (A) ATCC 15305, (C) 7108 and (E) 9325. Peptide Mass accuracy analyses. Peptide data were used to make the bar graph showing the accuracy of mass for peptides. (B) ATCC 15305, (D) 7108 and (F) 9325.
