# Supplementary material for: Staphylococcus saprophyticus Proteomic Analyses Elucidate Differences in the Protein Repertories among Clinical Strains Related to Virulence and Persistence
Source: Pathogens. 2020 Jan 19;9(1):69. doi: 10.3390/pathogens9010069 (PMC7169411; doi:10.3390/pathogens9010069)
Supplement: Supplementary file 1 [file pathogens-09-00069-s001.zip › New folder/Supplementary Table 1.pdf]

Supplementary Table 1 - List of proteins identified in this work.

Accession number1	Protein description	Log FC[2]			*p*-value[3]		
		ATCC vs 7108	ATCC vs 9325	7108 vs 9325	ATCC vs 7108	ATCC vs 9325	7108 vs 9325
	**Transcription**						
Q49UV9	Cold shock protein	-0.416	-0.224	0.192	0.794	0.849	0.912
Q49XK3	Cold shock protein CspA	-0.160	0.711	0.871	0.892	0.230	0.150
Q49V46	Transcription termination/antitermination protein NusG	0.227	-0.016	-0.243	0.616	0.966	0.560
Q49V52	DNA-directed RNA polymerase subunit beta	-0.050	0.497	0.547	0.993	0.756	0.775
Q49V53	DNA-directed RNA polymerase subunit beta	-0.096	-0.122	-0.025	0.963	0.939	1.000
Q49VL1	Probable transcriptional regulatory protein SSP2054	0.350	0.493	0.143	0.378	0.110	0.735
Q49VN3	Putative transcriptional regulator	-0.062	0.146	0.209	0.796	0.403	0.239
Q49WZ2	DNA-directed RNA polymerase subunit omega	-0.088	-0.088	0.000	0.689	0.609	1.000
Q49X40	GTP-sensing transcriptional pleiotropic repressor CodY	0.523	0.631	0.108	0.378	0.172	0.894
Q49X51	Transcription termination/antitermination protein NusA	0.240	-0.428	-0.668	0.796	0.534	0.335
Q49X65	Putative transcriptional regulator	0.361	0.098	-0.263	0.195	0.709	0.291
Q49Y04	RNA polymerase sigma factor SigA	-0.261	0.159	0.419	0.453	0.573	0.116
Q49Y48	Transcription elongation factor GreA	0.789	1.026	0.237	0.224	0.061	0.747
Q49Y64	Putative transcriptional regulator	-0.203	-0.225	-0.021	0.464	0.326	0.973
Q49ZE2	DNA-directed RNA polymerase subunit alpha	-0.047	-0.358	-0.311	0.954	0.364	0.467
Q49YJ2	HTH-type transcriptional regulator rot	0.280	-0.397	-0.677	0.706	0.500	0.239
**Amino acid metabolism**							
Q49V22	Cysteine synthase	-0.851	-0.593	0.258	0.000	0.007	0.248
Q49W45	Glycine cleavage system H protein	0.337	0.699	0.362	0.342	0.019	0.226
Q49XA2	Glutamine synthetase	-0.416	-0.885	-0.469	0.163	0.001	0.061
Q49YD9	Alanine dehydrogenase	0.879	2.013	1.134	0.183	0.001	0.042
Q49ZA2	Glutamine--fructose-6-phosphate aminotransferase [isomerizing]	-0.134	-2.288	-2.155	0.929	0.004	0.005
Q4A0E7	1-pyrroline-5-carboxylate dehydrogenase	1.054	1.308	0.254	0.082	0.017	0.724

Q49VA2	Branched-chain-amino-acid aminotransferase	-0.098	0.207	0.305	0.940	0.777	0.735
Q49VS0	Histidinol-phosphate aminotransferase	-0.196	0.098	0.294	0.644	0.778	0.405
Q49W97	Glutamate dehydrogenase	0.301	-0.041	-0.342	0.631	0.948	0.552
Q49YG8	DAHP synthetase-chorismate mutase	-1.122	-0.164	0.958	0.183	0.834	0.191
Q49YI1	D-alanine aminotransferase	0.524	0.752	0.228	0.464	0.172	0.762
Q49YI2	Putative dipeptidase SSP1012	-0.067	0.531	0.598	0.954	0.385	0.339
Q4A0D4	3-hydroxy-3-methylglutaryl coenzyme A reductase	0.119	0.732	0.613	0.901	0.147	0.253
Q4A0D6	3-hydroxy-3-methylglutaryl CoA synthase	0.063	-0.731	-0.794	0.938	0.067	0.050
**Protein synthesis**							
Q49UP8	Ribosome-binding ATPase YchF	1.159	1.167	0.008	0.070	0.038	1.000
Q49UZ9	Methionine--tRNA ligase	-0.214	1.851	2.065	0.901	0.044	0.024
Q49V37	Glutamate--tRNA ligase	0.418	1.593	1.175	0.602	0.013	0.058
Q49V49	50S ribosomal protein L10	-0.030	-0.586	-0.557	0.954	0.022	0.024
Q49V55	30S ribosomal protein S12	0.543	-0.411	-0.954	0.068	0.105	0.001
Q49V56	30S ribosomal protein S7	0.331	-0.543	-0.873	0.274	0.038	0.001
Q49X24	30S ribosomal protein S16	-0.050	-0.707	-0.656	0.933	0.022	0.027
Q49X41	30S ribosomal protein S2	0.397	-0.321	-0.718	0.274	0.316	0.019
Q49X44	Ribosome-recycling factor	0.442	-0.283	-0.725	0.082	0.195	0.001
Q49XT0	30S ribosomal protein S1	0.550	1.497	0.948	0.560	0.038	0.201
Q49Y85	50S ribosomal protein L21	0.523	-0.217	-0.740	0.224	0.573	0.042
Q49YB1	50S ribosomal protein L35	0.261	-1.160	-1.420	0.740	0.038	0.010
Q49YF2	30S ribosomal protein S4	0.557	-0.273	-0.830	0.059	0.311	0.002
Q49YU4	Glutamyl-tRNA(Gln) amidotransferase subunit A	-0.068	-0.657	-0.588	0.803	0.001	0.003
Q49ZE3	30S ribosomal protein S11	0.388	-0.908	-1.296	0.437	0.021	0.001
Q49ZE5	50S ribosomal protein L36	0.675	-0.656	-1.331	0.154	0.099	0.001
Q49ZE9	50S ribosomal protein L15	0.350	-0.403	-0.754	0.236	0.103	0.003
Q49ZF6	50S ribosomal protein L5	0.192	-0.332	-0.524	0.557	0.152	0.024
Q49ZG0	50S ribosomal protein L29	0.393	0.152	-0.241	0.038	0.385	0.168
Q49ZG1	50S ribosomal protein L16	0.109	-1.262	-1.371	0.938	0.063	0.048

Q49ZG2	30S ribosomal protein S3	0.045	-0.701	-0.746	0.901	0.001	0.000
Q49ZG4	30S ribosomal protein S19	0.816	-0.794	-1.611	0.030	0.021	0.000
Q49ZG5	50S ribosomal protein L2	0.103	-0.555	-0.659	0.760	0.021	0.005
Q49ZG6	50S ribosomal protein L23	0.317	-0.866	-1.182	0.263	0.001	0.000
Q49ZG7	50S ribosomal protein L4	0.184	-0.400	-0.583	0.591	0.116	0.024
Q49ZG9	30S ribosomal protein S10	0.351	-0.738	-1.089	0.197	0.002	0.000
Q49V57	Elongation factor G	0.414	-0.412	-0.826	0.082	0.047	0.000
Q49ZI5	Uncharacterized protein	-0.565	0.286	0.851	0.119	0.381	0.006
Q49UQ0	30S ribosomal protein S6	0.511	0.219	-0.292	0.245	0.585	0.507
Q49V10	50S ribosomal protein L25	-0.057	-0.434	-0.377	0.901	0.075	0.137
Q49V39	Cysteine--tRNA ligase	0.100	-0.122	-0.222	0.780	0.634	0.395
Q49V47	50S ribosomal protein L11	0.455	-0.189	-0.644	0.236	0.585	0.052
Q49V48	50S ribosomal protein L1	0.688	0.349	-0.339	0.439	0.634	0.713
Q49V50	50S ribosomal protein L7/L12	0.171	0.405	0.235	0.702	0.220	0.552
Q49VF3	Arginine--tRNA ligase	0.035	-0.732	-0.767	0.993	0.525	0.552
Q49WC5	Tryptophan--tRNA ligase	-0.060	0.132	0.192	0.954	0.815	0.780
Q49WL6	Peptide deformylase	0.211	0.411	0.200	0.559	0.120	0.536
Q49WX5	Isoleucine--tRNA ligase	0.986	0.087	-0.899	0.557	0.958	0.549
Q49X05	50S ribosomal protein L28	0.284	-0.014	-0.298	0.687	0.981	0.649
Q49X27	50S ribosomal protein L19	0.388	0.524	0.136	0.631	0.410	0.900
Q49X48	Proline--tRNA ligase	0.186	0.662	0.476	0.929	0.558	0.735
Q49X61	30S ribosomal protein S15	0.141	0.028	-0.113	0.633	0.920	0.713
Q49XR0	Asparagine--tRNA ligase	-0.220	-0.242	-0.022	0.892	0.777	1.000
Q49Y08	Glycine--tRNA ligase	-0.161	-0.663	-0.502	0.909	0.385	0.569
Q49Y27	30S ribosomal protein S20	0.022	-0.201	-0.223	0.963	0.480	0.446
Q49Y68	Aspartate--tRNA ligase	0.627	0.821	0.193	0.287	0.095	0.775
Q49Y83	50S ribosomal protein L27	0.543	0.218	-0.325	0.116	0.488	0.300
Q49YB0	50S ribosomal protein L20	-0.013	0.325	0.338	0.999	0.573	0.613

Q49YB4	Threonine--tRNA ligase	0.288	0.809	0.520	0.684	0.107	0.339
Q49YG2	Tyrosine--tRNA ligase	0.067	0.703	0.636	0.958	0.343	0.405
Q49YH3	Phenylalanine--tRNA ligase beta subunit	0.341	0.478	0.138	0.557	0.276	0.812
Q49YU3	Aspartyl/glutamyl-tRNA(Asn/Gln) amidotransferase subunit B	0.648	1.548	0.900	0.560	0.061	0.315
Q49YU5	Aspartyl/glutamyl-tRNA(Asn/Gln) amidotransferase subunit C	-0.048	-0.614	-0.566	0.945	0.067	0.096
Q49Z65	Peptide chain release factor 1	0.314	0.165	-0.149	0.631	0.767	0.833
Q49Z67	50S ribosomal protein L31 type B	0.359	0.190	-0.168	0.464	0.634	0.742
Q49ZD5	30S ribosomal protein S9	0.366	0.627	0.261	0.557	0.159	0.649
Q49ZD6	50S ribosomal protein L13	0.186	-0.634	-0.821	0.773	0.143	0.059
Q49ZE1	50S ribosomal protein L17	0.369	-0.243	-0.612	0.416	0.522	0.079
Q49ZF0	50S ribosomal protein L30	0.578	0.172	-0.405	0.081	0.573	0.169
Q49ZF1	30S ribosomal protein S5	0.174	-0.231	-0.405	0.760	0.581	0.334
Q49ZF2	50S ribosomal protein L18	-0.168	0.706	0.874	0.902	0.364	0.262
Q49ZF3	50S ribosomal protein L6	0.374	-0.149	-0.523	0.236	0.604	0.054
Q49ZF4	30S ribosomal protein S8	0.416	0.192	-0.225	0.537	0.727	0.735
Q49ZF7	50S ribosomal protein L24	0.213	0.297	0.084	0.703	0.500	0.906
Q49ZF8	50S ribosomal protein L14	0.547	-0.048	-0.595	0.233	0.920	0.134
Q49ZF9	30S ribosomal protein S17	0.318	-0.048	-0.367	0.439	0.900	0.268
Q49ZG3	50S ribosomal protein L22	0.657	0.037	-0.621	0.215	0.948	0.174
Q49ZG8	50S ribosomal protein L3	0.882	1.129	0.247	0.241	0.075	0.775
Q4A166	50S ribosomal protein L9	0.305	-0.067	-0.372	0.416	0.834	0.219
Q4A172	Serine--tRNA ligase	-1.280	0.035	1.315	0.123	0.966	0.061
Q49V58	Elongation factor Tu	0.018	0.251	0.233	0.969	0.343	0.391
Q49X42	Elongation factor Ts	0.318	0.182	-0.136	0.269	0.482	0.664
Q49XX3	Elongation factor P	0.101	-0.238	-0.339	0.901	0.604	0.505
**Protein Folding and degradation**							
Q49Y22	Chaperone protein DnaK	0.407	-0.300	-0.707	0.060	0.107	0.000

Q49Y23	Protein GrpE	0.476	0.063	-0.413	0.015	0.752	0.020
Q49YY6	10 kDa chaperonin	-0.195	1.099	1.294	0.601	0.000	0.000
Q49WT1	Uncharacterized protein SSP1625	-0.216	-2.604	-2.387	0.467	0.000	0.000
Q49VZ2	ATP-dependent Clp protease proteolytic subunit	0.035	0.641	0.606	0.963	0.135	0.174
Q49YS4	Leucyl aminopeptidase	0.527	0.453	-0.073	0.559	0.529	0.965
Q49W93	Putative peptidyl-prolyl cis-trans isomerase	0.843	0.743	-0.099	0.117	0.099	0.900
Q49YA6	ATP-dependent Clp protease ATP-binding subunit ClpX	-0.278	0.657	0.934	0.796	0.391	0.225
Q49YA7	Trigger factor	0.081	-0.218	-0.300	0.796	0.345	0.187
Q49YY5	60 kDa chaperonin	0.150	0.358	0.209	0.602	0.101	0.390
**'de novo' Pyrimidine nucleobase biosynthetic process**							
Q49VB7	3-hexulose-6-phosphate synthase 3	-0.303	-0.952	-0.648	0.560	0.021	0.104
Q49WT0	3-hexulose-6-phosphate synthase 2	-0.833	-0.086	0.747	0.005	0.777	0.006
Q49X43	Uridylate kinase	0.072	0.625	0.553	0.901	0.034	0.055
Q49XT1	Cytidylate kinase	0.419	0.365	-0.054	0.284	0.287	0.922
Q49Y11	Cytidine deaminase	-0.406	-0.462	-0.055	0.274	0.136	0.912
Q49Z59	Uracil phosphoribosyltransferase	-0.457	0.084	0.542	0.378	0.849	0.201
Q49Z73	CTP synthase	0.987	-0.110	-1.097	0.426	0.925	0.271
**"de novo" purine metabolism**							
Q49WI9	N5-carboxyaminoimidazole ribonucleotide synthase PurK	2.377	2.377	0.000	0.000	0.000	1.000
Q49WJ0	Phosphoribosylaminoimidazole-succinocarboxamide synthase PurC	2.184	2.184	0.000	0.000	0.000	1.000
Q49WJ1	Phosphoribosylformylglycinamidine synthase subunit PurS	1.035	1.035	0.000	0.000	0.000	1.000
Q49WJ2	Phosphoribosylformylglycinamidine synthase subunit PurQ	1.006	1.006	0.000	0.011	0.007	1.000
Q49WJ3	Phosphoribosylformylglycinamidine synthase subunit PurL	3.345	3.345	0.000	0.000	0.000	1.000
Q49WJ5	Phosphoribosylformylglycinamidine cyclo-ligase PurM	2.365	2.365	0.000	0.000	0.000	1.000
Q49WJ7	Bifunctional purine biosynthesis protein PurH	4.249	4.249	0.000	0.000	0.000	1.000
Q49WJ8	Phosphoribosylamine--glycine ligase PurD	3.038	3.038	0.000	0.000	0.000	1.000
Q49UU8	Inosine-5'-monophosphate dehydrogenase	0.129	0.487	0.358	0.910	0.450	1.000

Q49UU9	GMP synthase [glutamine-hydrolyzing]	0.780	-0.541	-1.321	0.458	0.529	0.104
Q49V19	Putative hypoxanthine-guanine phosphoribosyltransferase	0.109	-0.330	-0.439	0.892	0.452	0.322
Q49XU7	Putative ADP-ribose pyrophosphatase	-0.068	0.093	0.160	0.892	0.746	0.619
Q49V09	Ribose-phosphate pyrophosphokinase	-0.532	-0.167	0.365	0.591	0.838	0.717
Q49XS3	Nucleoside diphosphate kinase	0.225	0.304	0.079	0.326	0.099	0.753
Q49WI8	N5-carboxyaminoimidazole ribonucleotide mutase	-0.068	-0.068	0.000	0.740	0.659	1.000
**Glycolysis**							
Q49WL8	Pyruvate dehydrogenase E1 component alpha subunit	-0.043	-0.662	-0.620	0.922	0.005	0.006
Q49VZ9	Glyceraldehyde-3-phosphate dehydrogenase	0.319	-0.257	-0.575	0.218	0.243	0.008
Q49W00	Phosphoglycerate kinase	0.128	-0.622	-0.750	0.602	0.002	0.000
Q49W01	Triosephosphate isomerase	0.431	-0.399	-0.830	0.068	0.053	0.000
Q49W02	2_3-bisphosphoglycerate-independent phosphoglycerate mutase	0.330	-0.386	-0.716	0.224	0.086	0.002
Q49YC7	Pyruvate kinase	0.142	-0.373	-0.515	0.591	0.061	0.010
Q49ZZ2	2_3-bisphosphoglycerate-dependent phosphoglycerate mutase	0.268	1.226	0.958	0.631	0.006	0.024
Q49W03	Enolase	0.183	0.014	-0.168	0.557	0.961	0.527
Q49WA1	Glucose-6-phosphate isomerase	0.444	0.642	0.198	0.581	0.311	0.811
Q49WL9	Pyruvate dehydrogenase E1 component beta subunit	-0.116	-0.360	-0.245	0.687	0.079	0.264
Q49WM0	Dihydrolipoamide acetyltransferase component of pyruvate dehydrogenase complex	0.227	1.206	0.980	0.840	0.077	0.169
Q49Z72	Fructose-bisphosphate aldolase	0.045	-0.127	-0.172	0.916	0.585	0.506
Q4A0Q6	Fructose-bisphosphate aldolase class I	0.553	-0.042	-0.596	0.128	0.917	0.058
**Lactose metabolism**							
Q49VP7	Tagatose-6-phosphate kinase	1.526	0.952	-0.574	0.005	0.051	0.264
**Lactic fermentation**							
Q49YJ3	L-lactate dehydrogenase	1.148	0.293	-0.855	0.195	0.727	0.271
**One carbon metabolism**							
Q49WI7	Bifunctional protein FolD	2.914	2.839	-0.075	0.000	0.000	0.834
Q49Z60	Serine hydroxymethyltransferase	0.624	0.214	-0.410	0.012	0.364	0.063

**Phosphoenolpyruvate-dependent sugar phosphotransferase system (PTS)**							
Q49WK7	Phosphoenolpyruvate-protein phosphotransferase	0.321	-0.553	-0.874	0.236	0.021	0.000
Q49ZA5	Phosphotransferase system mannitol-specific IIA domain	-0.526	-0.526	0.001	0.053	0.033	1.000
**Glycerol Metabolic Process**							
Q49X93	Glycerol kinase	0.699	-0.468	-1.167	0.197	0.333	0.011
**Pentose Phosphate Pathway**							
Q49XD6	Transketolase	0.221	-0.478	-0.699	0.439	0.034	0.002
Q49XV6	6-phosphogluconate dehydrogenase_ decarboxylating	0.099	-0.402	-0.501	0.741	0.057	0.018
Q49XV2	Glucose-6-phosphate 1-dehydrogenase	0.298	0.437	0.139	0.773	0.563	0.907
Q49YL0	Transaldolase	-0.011	-0.346	-0.335	0.986	0.085	0.104
Q49Z84	Deoxyribose-phosphate aldolase	0.173	0.196	0.023	0.898	0.790	1.000
**Carbohydrate Metabolic process**							
Q49ZA7	Phosphoglucosamine mutase	0.869	1.566	0.697	0.378	0.044	0.407
Q49Z82	Phosphopentomutase	-1.769	-1.167	0.603	0.011	0.061	0.377
Q49Z69	Putative aldehyde dehydrogenase SSP0762	-0.226	-1.596	-1.370	0.786	0.007	0.016
Q4A113	Putative UDP-glucose 6-dehydrogenase	0.569	0.569	0.000	0.113	0.061	1.000
Q4A122	UTP--glucose-1-phosphate uridylyltransferase 1	-0.016	-0.016	0.000	0.954	0.919	1.000
**Fatty acid metabolism**							
Q49WB7	3-oxoacyl-[acyl-carrier-protein] synthase 2	0.130	-0.495	-0.625	0.696	0.044	0.011
Q49X16	Acyl carrier protein	0.644	-0.055	-0.699	0.057	0.867	0.018
Q4A0D5	Acetyl-CoA acetyltransferase	-0.214	-1.708	-1.495	0.782	0.002	0.005
Q49WM1	Dihydrolipoyl dehydrogenase	0.174	-0.394	-0.568	0.557	0.068	0.010
Q49UH6	Putative acyl-CoA hydrolase	0.376	0.165	-0.211	0.456	0.704	0.678
Q49VJ6	Putative dihydroxyacetone kinase	0.324	0.171	-0.153	0.279	0.529	0.640
Q49WB6	3-oxoacyl-[acyl-carrier-protein] synthase 3	0.151	-0.680	-0.831	0.901	0.311	0.215
Q49WE0	Enoyl-[acyl-carrier-protein] reductase [NADPH]	-0.290	-0.232	0.058	0.631	0.634	0.952
Q49X15	3-oxoacyl-[acyl-carrier protein] reductase	0.380	0.279	-0.101	0.594	0.633	0.911

Q49YC9	Acetyl-coenzyme A carboxylase carboxyl transferase subunit alpha	-0.070	0.000	0.070	0.782	1.000	0.764
Q49YE2	Acetate kinase	0.804	0.517	-0.287	0.462	0.572	0.801
**Tricarboxylic Acid Cycle**							
Q49XE4	Aconitate hydratase	-1.895	2.000	3.900	0.096	0.044	0.000
Q49XM4	Dihydrolipoyllysine-residue succinyltransferase component of 2-oxoglutarate dehydrogenase complex	0.607	1.615	1.010	0.462	0.014	0.117
Q49YC4	Isocitrate dehydrogenase [NADP]	-0.251	2.022	2.270	0.786	0.002	0.001
Q49X32	Succinate--CoA ligase [ADP-forming] subunit beta	0.962	1.164	0.202	0.233	0.084	0.834
Q49X33	Succinate--CoA ligase [ADP-forming] subunit alpha	0.959	0.617	-0.343	0.127	0.262	0.612
Q49YC5	Citrate synthase	0.237	0.147	-0.090	0.714	0.777	0.909
Q4A102	Probable malate:quinone oxidoreductase	0.370	-0.076	-0.446	0.776	0.948	0.707
**ATP synthesis**							
Q49Z50	ATP synthase subunit beta	-0.061	-0.378	-0.317	0.796	0.026	0.056
Q49W80	NADH dehydrogenase-like protein SSP1834	0.606	2.729	2.123	0.467	0.000	0.002
Q49YW3	Probable manganese-dependent inorganic pyrophosphatase	0.372	-0.059	-0.431	0.175	0.815	0.060
Q49Z51	ATP synthase gamma chain	-0.190	0.105	0.295	0.594	0.727	0.314
Q49Z52	ATP synthase subunit alpha	-0.186	-0.418	-0.232	0.557	0.061	0.335
**Vitamins and cofactors**							
Q49VD0	Putative phosphomethylpyrimidine kinase	-0.423	-0.282	0.141	0.389	0.497	0.787
Q49XG3	Probable tautomerase SSP1389	-0.209	0.004	0.213	0.560	0.992	0.506
Q49Y99	Glutamate-1-semialdehyde 2_1-aminomutase 2	0.101	0.300	0.198	0.929	0.633	0.801
Q49YA0	Delta-aminolevulinic acid dehydratase	0.084	0.084	0.000	0.570	0.482	1.000
Q49YJ6	6_7-dimethyl-8-ribityllumazine synthase	0.602	-0.156	-0.758	0.209	0.727	0.058
**Cell wall synthesis and remodeling**							
Q49W64	Lipoyl synthase	0.223	0.851	0.628	0.602	0.013	0.058
Q49W71	D-alanine--poly(phosphoribitol) ligase subunit 1	-1.709	1.228	2.937	0.008	0.038	0.000
Q49W73	D-alanine--poly(phosphoribitol) ligase subunit 2	-1.327	-0.597	0.730	0.013	0.220	0.141

Q4A0G5	Probable transglycosylase IsaA	2.075	0.681	-1.394	0.016	0.403	0.075
Q49VI7	Teichoic acid biosynthesis protein	-0.020	0.056	0.076	0.954	0.777	0.753
Q4A0A8	Ribitol-5-phosphate cytidylyltransferase	0.543	0.584	0.041	0.128	0.059	0.949
Q49Z31	D-alanine--D-alanine ligase	0.407	0.498	0.091	0.557	0.356	0.912
**DNA Binding Protein**							
Q49YP0	Uncharacterized protein	0.103	-0.767	-0.870	0.892	0.049	0.024
**DNA repair**							
Q49Y00	Probable endonuclease 4	0.408	0.404	-0.004	0.263	0.183	1.000
**Cell division**							
Q49WW8	Cell division protein FtsZ	-0.312	0.224	0.536	0.705	0.655	0.339
Q49WX4	Putative cell-division initiation protein	0.387	0.161	-0.226	0.727	0.464	0.678
Q49XS7	Bacterial nucleoid DNA-binding protein HU	-0.280	-0.385	-0.104	0.458	0.181	0.790
Q49ZB1	Iron-sulfur cluster carrier protein	0.002	0.157	0.156	1.000	0.522	0.569
**Cell signaling**							
Q49ZE7	Adenylate kinase	0.318	-0.435	-0.753	0.195	0.038	0.001
Q49YM8	Signal transduction protein	0.666	0.791	0.124	0.037	0.007	0.743
**Transport**							
Q49WK6	Phosphocarrier protein HPr	-0.079	0.278	0.357	0.892	0.364	0.245
Q49XN3	PTS system glucose-specific IIA component	0.000	-0.120	-0.119	1.000	0.683	0.739
Q49XJ3	ATPase component of ABC transporter with duplicated ATPase domains	1.377	0.995	-0.382	0.051	0.100	0.622
**Oxidative stress**							
Q49UT8	Alkyl hydroperoxide reductase subunit C	0.490	-0.408	-0.899	0.026	0.040	0.000
Q49UU5	Nitronate monooxygenase	-0.339	1.063	1.402	0.402	0.001	0.000
Q49W43	Putative thioredoxin	0.176	0.574	0.398	0.644	0.047	0.182
Q49WR2	Thioredoxin	0.494	-0.133	-0.627	0.011	0.480	0.001

Q49XC1	Catalase	3.691	3.811	0.120	0.000	0.000	0.735
Q49XN4	Peptide methionine sulfoxide reductase MsrB	0.228	0.460	0.232	0.449	0.044	0.339
Q49XZ6	Superoxide dismutase [Mn/Fe]	0.338	-0.406	-0.744	0.128	0.038	0.000
Q49YE0	Putative universal stress protein SSP1056	2.055	2.055	0.000	0.000	0.000	1.000
Q49YE4	Probable thiol peroxidase	0.280	-0.419	-0.699	0.376	0.085	0.005
Q4A052	Putative glyoxalase family protein	-0.077	-0.375	-0.298	0.922	0.367	0.530
Q4A054	Putative glyoxalase family protein	-0.105	-0.302	-0.198	0.853	0.385	0.647
**Iron metabolism**							
Q49UL6	Flavohemoprotein	0.628	1.161	0.533	0.222	0.009	0.239
Q49WI6	Putative flavohemoprotein	1.422	-0.549	-1.971	0.130	0.529	0.016
Q49YT7	Bacterial non-heme ferritin	0.428	-0.657	-1.085	0.496	0.168	0.024
Q49ZK8	Putative ABC-type cobalamin Fe3+-siderophores transport system periplasmic component	1.052	-1.038	-2.090	0.233	0.161	0.006
Q49VE0	Putative heme-dependent peroxidase SSP2125	1.522	1.043	-0.479	0.003	0.026	0.331
Q49VT4	Putative ABC-type cobalamin Fe3+-siderophores transport system periplasmic component	0.591	0.300	-0.292	0.557	0.717	0.769
Q49YM6	Ferrochelatase	0.406	0.174	-0.232	0.321	0.633	0.567
**Nitrogen metabolism**							
Q4A0J5	Urease subunit alpha	-1.782	1.338	3.120	0.034	0.068	0.000
Q4A0J8	Urease accessory protein UreG	-0.416	0.936	1.352	0.464	0.038	0.003
**Stress response**							
Q49V34	ATP-dependent Clp protease ATP-binding subunit ClpC	0.285	0.378	0.093	0.760	0.583	0.939
Q49W40	Organic hydroperoxide resistance protein-like 1	0.000	-0.148	-0.148	1.000	0.319	0.334
Q49YD5	Putative universal stress protein	-0.730	-0.638	0.092	0.186	0.166	0.907
Q49YH5	Putative thioredoxin	-0.033	-0.124	-0.092	0.958	0.743	0.860
Q49YY9	Nitroreductase family protein	0.112	0.153	0.040	0.602	0.381	0.885
Q49ZC7	Alkaline shock protein 23	0.000	-0.346	-0.346	1.000	0.183	0.206
Q49ZX5	Putative small heat shock protein	0.175	0.017	-0.158	0.646	0.963	0.678
**Uncharacterized Protein**							

Q49Y55	UPF0297 protein SSP1144	-0.526	-0.583	-0.058	0.088	0.034	0.900
Q49YH0	UPF0478 protein SSP1024	-0.434	0.271	0.706	0.197	0.364	0.014
Q49YS0	Uncharacterized protein SSP0918	0.000	-0.327	-0.327	1.000	0.044	0.045
Q49ZY4	Uncharacterized protein	-0.128	-0.408	-0.280	0.602	0.037	0.152
Q49WA8	Uncharacterized protein SSP1806	0.350	-0.646	-0.996	0.557	0.135	0.023
Q49XB9	Uncharacterized protein	0.261	-0.769	-1.030	0.591	0.038	0.006
Q49W20	Putative lysophospholipase	-0.567	-0.681	-0.114	0.128	0.038	0.794
Q4A0W6	UPF0176 protein SSP0127	0.731	0.731	0.000	0.012	0.007	1.000
Q49V17	Putative RNA binding protein	0.201	0.070	-0.131	0.559	0.801	0.700
Q49VE1	Phosphotransacetylase	0.377	-0.247	-0.624	0.342	0.480	0.054
Q49VG6	Uncharacterized protein	0.164	-0.345	-0.510	0.689	0.261	0.090
Q49VT0	Ribonucleoside-diphosphate reductase minor subunit	0.529	0.731	0.202	0.455	0.169	0.780
Q49VV4	Uncharacterized protein SSP1961	-0.529	0.000	0.529	0.119	1.000	0.066
Q49W17	UPF0337 protein SSP1897	-0.067	-0.465	-0.398	0.958	0.529	0.659
Q49W44	Uncharacterized protein	-0.088	0.344	0.432	0.922	0.480	0.377
Q49W54	Uncharacterized protein	0.494	0.598	0.104	0.570	0.385	0.926
Q49WE4	Putative phosphoesterase SSP1770	0.525	0.429	-0.096	0.402	0.398	0.907
Q49WH5	Uncharacterized protein	0.167	0.041	-0.126	0.572	0.867	0.664
Q49WL4	UPF0356 protein SSP1700	-0.787	0.218	1.005	0.378	0.777	0.169
Q49X06	Uncharacterized protein	0.260	0.421	0.161	0.692	0.403	0.806
Q49X75	Uncharacterized protein	0.000	0.308	0.308	1.000	0.070	0.072
Q49XA3	Uncharacterized protein	0.208	-0.470	-0.678	0.692	0.229	0.078
Q49XP1	Uncharacterized protein	0.040	-0.537	-0.577	0.958	0.168	0.154
Q49XV9	UPF0403 protein SSP1241	-0.061	-0.450	-0.388	0.922	0.161	0.254
Q49XY9	Uncharacterized protein	0.314	0.005	-0.308	0.165	0.981	0.111
Q49Y63	UPF0337 protein SSP1134	-0.080	-0.607	-0.527	0.963	0.573	0.690
Q49YI7	Uncharacterized protein	0.305	0.101	-0.205	0.456	0.777	0.595
Q49YN8	UPF0342 protein SSP0954	0.488	0.364	-0.124	0.471	0.528	0.894
Q49YQ1	Uncharacterized protein	0.248	0.133	-0.115	0.699	0.797	0.885

Q49YS1	Uncharacterized protein	-0.233	-0.741	-0.508	0.731	0.116	0.328
Q49YS5	UPF0435 protein SSP0913	-0.038	-0.364	-0.326	0.954	0.302	0.375
Q49Z85	Purine nucleoside phosphorylase DeoD-type	0.291	0.021	-0.271	0.581	0.966	0.569
Q49Z99	Putative hydrolase	-0.281	0.146	0.426	0.464	0.648	0.169
Q49ZB5	Probable uridylyltransferase SSP0716	0.294	0.630	0.336	0.780	0.398	0.733
Q49ZM5	Putative 2-hydroxyacid dehydrogenase SSP0606	-0.017	-0.415	-0.398	0.992	0.356	0.390
Q49ZQ3	Putative short chain dehydrogenase	0.000	0.027	0.027	1.000	0.848	0.900
Q4A0P1	Putative nitroreductase	0.168	0.258	0.090	0.560	0.242	0.759
Q4A0Y2	Uncharacterized protein	-0.041	-0.777	-0.735	0.992	0.482	0.552

[1] Accession number provided by Uniprot Database (http://www.uniprot.org/).

[2] Obtained from limma's topTable by subtracting the average expression in log2 scale against the strains.

[3] p-value from the Student's t distribution. Proteins with p-value ≤ 0.05 were considered regulated among the strains.
